# Highly Flexible and Foldable Paper-Based Thermoelectric Generator Prepared with Post-Treatment-Free PEDOT:PSS Hybrid Ink

**DOI:** 10.3390/polym15214215

**Published:** 2023-10-25

**Authors:** Guixiang Chen, Zhenhang He, Zhen Liu, Xin Li, Zhengyin Yao, Peng Zhang

**Affiliations:** Key Laboratory for Polymeric Composite and Functional Materials of Ministry of Education, School of Materials Science and Engineering, Sun Yat-sen University, Guangzhou 510275, China; chengx57@mail2.sysue.du.cn (G.C.); hezhh33@mail2.sysu.edu.cn (Z.H.); liuzh287@mail.sysu.edu.cn (Z.L.); lixin266@mail2.sysu.edu.cn (X.L.); yaozhy23@mail2.sysu.edu.cn (Z.Y.)

**Keywords:** paper-based thermoelectric, PEDOT:PSS, hierarchical structure, mechanical stability

## Abstract

Paper-based thermoelectric (PTE) generators have recently emerged as a green technology that can help alleviate environment pollution and the energy crisis. In this work, a PTE generator was prepared by coating a post-treatment-free thermoelectric ink consisting of poly(3,4-ethylenedioxythiophene)/polystyrene sulfonate (PEDOT:PSS) doped with 1-ethyl-3-methylimidazolium:tricyanomethanide (EMIM:TCM) onto the card paper. By tuning the molar concentration of the EMIM:TCM to 0.17 M and with hot-pressing, the PTE generator showed a decent power factor (PF) value of 6.82 μW m^−1^ K^−2^, which was higher than the values of PTE in the literature. This phenomenon could be attributed to the synergistic effect of high-performance thermoelectric ink (i.e., PF = 175 μW m^−1^ K^−2^ when deposited on glass slide) and the hot-pressing. The hot-pressing enhanced the packing density of cellulose fibers and the associated PEDOT:PSS hybrid, which enabled the formation of long-range conductive paths. In addition, the PTE had good mechanical stability, indicated by no significant change of the power factor values after cyclic folding 10,000 times. Moreover, the structure of as-prepared PTE could be easily tuned into different shapes that are promising for the preparation of flexible wearable thermoelectric generators.

## 1. Introduction

In recent years, intelligent wearable electronic devices have received increasing attention. For a portable use of wearable devices, there have been numerous studies on integrated energy harvesters, such as piezo-electric batteries [1,2], moisture-driven power generators [3], and thermoelectric generators (TEGs). Among these devices, TEGs are a kind of green energy source, which could directly convert waste heat into useful electricity that can power wearable electronics and the “Internet of Things” [4]. Especially, flexible TEGs that could easily adapt to the complex geometry and compliant material property of skin have attracted lots of interests [4,5]. In the past decade, great progress has been achieved in the development of flexible wearable TEGs, such as high-performance thermoelectric inks [5,6], post-treatment-free thermoelectric inks [4], flexible substrates [5], and coating technologies [7,8,9,10], among which, poly(3,4-ethylenedioxythiophene):polystyrene sulfonate (PEDOT:PSS) and paper have evolved as the promising thermoelectric ink material and substrate, respectively, mainly due to their unique features in superior biocompatibility, high foldability, and good adaptability to the wet-coating process [11,12,13].

PEDOT:PSS is a charged polymer complex consisting of conductive and hydrophobic PEDOT, and insulating and hydrophilic PSS chains, which bond together via columbic interactions. It is one of the most successfully commercialized conjugated polymers that is widely used in flexible electronics, organic electronics, conductive coatings, and photovoltaics [14,15,16] because PEDOT:PSS has good aqueous dispersibility/stability, ease processability, high conductivity, superior flexibility and stretchability, and excellent biocompatibility. These features endow PEDOT:PSS with not only good thermoelectric performance (e.g., typical figure of merit of 0.42 [12]) but also the capability to be fabricated into free-standing and foldable sheets after being coated onto the polymer substrates [5,17,18]. Paper substrates are among the most promising candidates for flexible, inexpensive, and environmentally friendly TEGs [8,9,13]. Previous works have demonstrated that PEDOT:PSS/paper TEGs could integrate the thermoelectric functionality of PEDOT:PSS with the merits of the paper substrate via simple and cost-effective wet-coating protocols. For example, Jiang et al. [19]. and Wei et al. [20]. have independently demonstrated that the functional PEDOT:PSS ink could be coated onto paper substrate to fabricate free-standing PEDOT:PSS/paper TEGs, via writing or screen-printing, respectively. Moreover, Deng et al. reported that the PEDOT:PSS/paper TEGs showed excellent mechanical stability, i.e., thermoelectric performance was kept almost stable after 120 folding cycles [21]. It should be pointed out that boosting the mechanical properties, such as the stretchability/foldability, of PEDOT:PSS/paper is also important to enhance the overall performance and stability of their F-TEGs [5]. However, the thermoelectric performance of the PEDOT:PSS/paper hybrid was generally much lower that the counterparts prepared by depositing the PEDOT:PSS on traditional substrates like glass slides or plastic foils.

By addressing this issue, we fabricated a PTE generator by coating the PEDOT:PSS hybrid ink onto the playing card paper substrate and conducted a systematic study of structure–property correlation and mechanical stability. To enhance the thermoelectric performance of PEDOT:PSS, a 1-ethyl-3-methylimidazolium:tricyanomethanide (EMIM:TCM) ionic liquid was employed as a secondary dopant to develop a high-performance thermoelectric ink [4,6]. The PEDOT:PSS/EMIM:TCM/paper (PPETn-P, where n indicates the molar concentration of EMIM:TCM) was post-treated with hot-pressing to enhance the packing density of the cellulose fiber and PEDOT:PSS. The PEDOT:PSS/EMIM:TCM/glass (PPET-G), prepared by coating onto glass slides, was included as a standard for comparison with PPET-P. The surface morphology and crystalline behavior of the hot-pressed PPET-P film were characterized with scanning electron microscopy (SEM), optical microscopy (OM), and grazing-incidence X-ray diffraction (GIXRD), respectively. Moreover, the folding and stretchability of the PPET-GTE were characterized with the folding tester and tensile testing machine, respectively, based on which, the decent thermoelectric performance and excellent mechanical stability of PPET-P was rationalized via the structure change and associated molecular interactions. This fundamental knowledge could illuminate the design and fabrication of the paper-based wearable electronics.

## 2. Experimental Section

### 2.1. Materials

PEDOT:PSS aqueous dispersion (Clevios PH 1000) was purchased from Heraeus (Leverkusen, Germany). Clevios PH 1000 had a solid content of 1.3 wt% PEDOT:PSS, and the weight ratio of PSS to PEDOT was 2.5. 1-ethyl-3-methylimidazolium tricyanomethanide (EMIM:TCM) was purchased from TCI Chemical Industry Development Co., Ltd. (Shanghai, China). Distilled water was purchased from Watsons (Guangzhou, China). Ethylene glycol (EG) was obtained from Aladin Co., Ltd. (Shanghai, China). Playing cards (type 959) were purchased from Yaoji Playing Cards Co., Ltd. (Shanghai, China). Glass slides (size: 24 mm × 24 mm) were purchased from Aladdin Bio-Chem Technology Co., Ltd. (Shanghai, China). The silicon wafer ((100) orientation) was purchased from PlutoSemi Co., Ltd. (Foshan, China) and used after cutting it into small pieces.

### 2.2. Sample Preparation

The post-treatment-free TE ink was prepared by adding the EMIM:TCM into the PEDOT:PSS aqueous dispersion at a set of molar concentration values, i.e., *n* = 0, 0.10, 0.15, 0.17, 0.20, and 0.30 M. The PEDOT:PSS/EMIM:TCM (PPET*_n_*) mixture was homogenized by magnetically stirring for 6 h at room temperature before the coating experiment. The as-prepared ink was drop-casted onto the paper or glass slide. The laboratory environment was 25 °C and 70 RH%. The purple core layer of the playing card was used for the matrix of the PPET. For the matrix preparation, the card was first immersed in EG for 24 h, to detach the core layer from the adjacent top and bottom layers of the card. Afterwards, the wet matrix was dried at 50 °C for 1 h with a laboratory oven, followed by cutting it into small pieces of 2 × 3 cm^2^ with a sharp knife.

The PPET-P composite was prepared by coating the PPET aqueous dispersion onto the paper matrix. First, 0.9 mL PPET aqueous dispersion was coated onto a piece of paper matrix by drop-casting with a pipette. Second, the wet sample was dried at 50 °C for 1 h with a laboratory oven. Finally, the sample was hot-pressed with a machine (ZS-406BE-30-310, Zhuosheng Machinery Equipment Co., Ltd., Dongguan, China) at 130 °C and 8 MPa for 30 min. The as-prepared PPET-P composite was cut into the desired shapes for the assembly of the wearable TEG device, and the thin copper wire and commercial silver paste were used as the electrode of the device.

### 2.3. Sample Characterization

Electrical conductivity of the sample was measured using the four-point probe method with a digital multimeter (RTS-8, Guangzhou Sitanpu Technology Co., Ltd., Guangzhou, China). The Seebeck coefficient was calculated from the linear correlation between the voltage generated by the thermoelectric effect and the temperature gradient across two silver paste dots that were deposited on the sample, as shown in the Figure S3. The dots had a diameter of 1 mm and were 6 mm separated from each other. A homemade Peltier heater was utilized to control the sample temperature, and a Fluke 8846A digital multimeter was used to record the thermoelectric voltage. For the *σ* and *S* values, each measurement was repeated 5 times, and the averaged value was used in this work.

The surface morphology of the PPET-P sample was characterized using both optical microscopy (OM) and scanning electron microscopy (SEM). For the OM measurement, a Leica DM2700M microscope (Wetzlar, Germany) was used, with the magnification set at 500× and working in reflection mode. For the SEM measurement, a cold field-emission scanning electron microscope (S-4800, Hitachi, Tokyo, Japan) was used. Moreover, the crystalline structure of the sample was measured with X-ray diffraction in grazing-incident mode (GIXRD) by fixing the sample on the silicon substrate. The GIXRD experiments were undertaken using a Bruker D8 Advance (Bruker, Germany) setup, with the grazing incident angle (*α_i_*), X-ray wavelength, working current and voltage set at 2.0°, 1.54 Å, 40 mA, and 40 kV, respectively. The folding property of the sample was undertaken with a home-made setup, which controlled the folding angle (*β*) of the composite sample by set the position of the sliders. For the *σ* and *S* values of the cyclically folded samples, each measurement was repeated five times, and the averaged value was used in this work. The stretching property of the sample was characterized using a computerized servo material testing machine (HZ-1007C, Dongguan LiXian Instrument Technology Co., Ltd., Dongguan, China) with a 1BB-type sample of GB/T 1040.1-2018 [22].

## 3. Results and Discussion

The performance of PEDOT-based TEGs is evaluated by the power factor (PF) value, and PF = *σ* × *S*^2^, where *σ* and *S* represent the samples’ electrical conductivity and Seebeck coefficient, respectively [23]. This could be attributed to the fact that the polymer materials usually have a intrinsically low thermal conductivity value that is difficult to measure. The conversion mechanism of TEGs was demonstrated in the Figure S1. In this work, the PEDOT:PSS doped with EMIM:TCM (i.e., PPET) was chosen as the study subject due to the following considerations. Firstly, the PPET could be dispersed in water to form stable aqueous dispersion that would be suitable for the wet-coating process, and the simple drop-casting method was used in this work. Secondly, the doping with EMIM:TCM would greatly improve (~1000 times higher) the thermoelectric performance of PEDOT:PSS mainly due to the significant enhancement of the sample’s conductivity while maintaining the Seebeck coefficient value [4,6]. Most importantly, the dried PPET-G sample showed a good thermoelectric performance (PF = 175 μW m^−1^ K^−2^ [6]) without post-treatment, which usually involves the application of a toxic organic solvent and cumbersome processing procedure.

**Figure 1:**
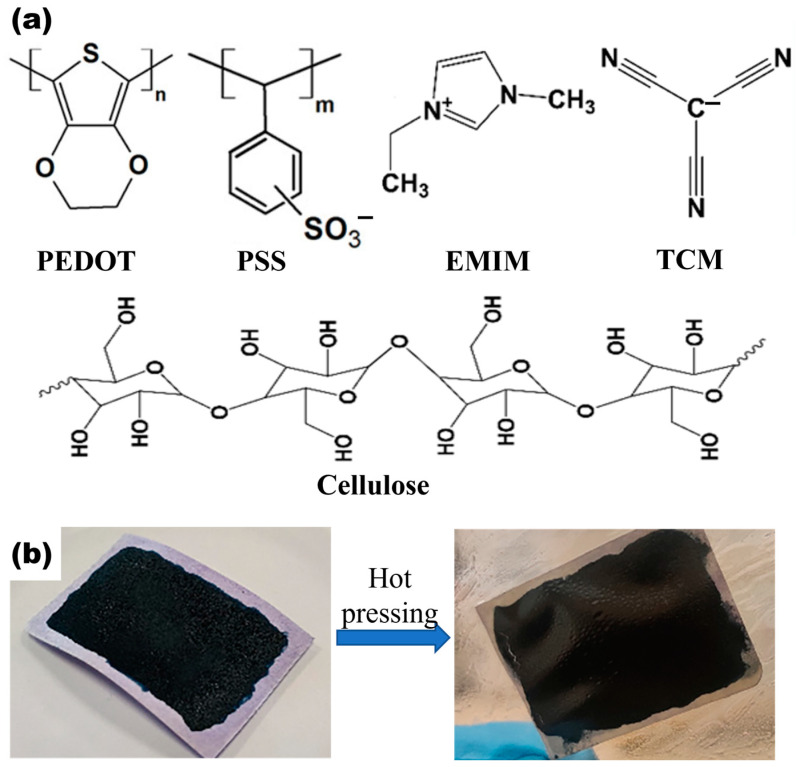
Design and preparation of PPET-P. (**a**) Chemical structure of PEDOT:PSS, EMIM:TCM, and cellulose. (**b**) Photo images of the drop-casted PPET-P samples before and after hot-pressing.

Figure 1a shows the molecular structure of the PEDOT:PSS, EMIM:TCM, and cellulose (the primary structural element and the most important component of paper). The drop-casting method was used to deposit the PPET aqueous dispersion on paper (PPET-P) and glass (PPET-G) substrates, respectively. Figure 1b shows the photo image of the dried PPET-P before and after hot-pressing. It should be noted that hot-pressing was employed to improve the thermoelectric performance of the PPET-P. Similar strategy has been applied by Palaporn et al. [24] and Gao et al. [25], who independently demonstrated that the hot-pressing could enhance the thermoelectric performance of the paper-based TEGs by improving the samples’ microstructure.

**Figure 2:**
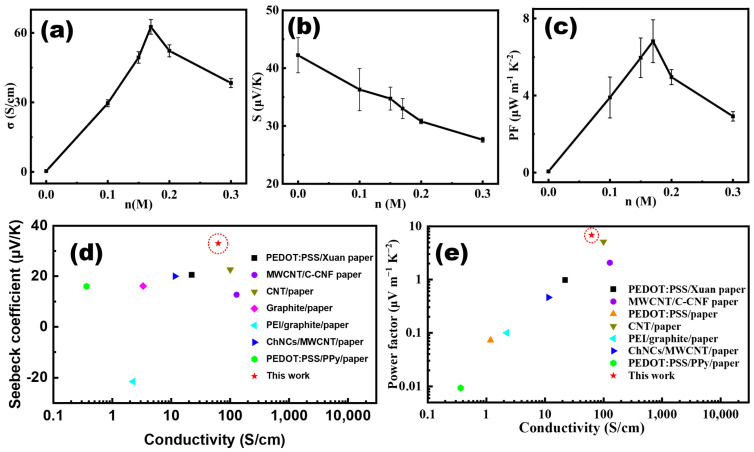
Performance of hot-pressed PPET*_n_*-P. (**a**–**c**) *σ*, S, and PF of PPET-P as a function of the EMIM:TCM molar concentration (*n*). Comparison with the literature reported (**d**) *S* and (**e**) PF values of paper-based thermoelectric materials [21,27,28,29,30,31,32]. In the panel (**d**,**e**), the *S* and PF values of this work, represented by a red star, was highlighted with a red dashed-line circle.

Figure 2a shows that the electrical conductivity (*σ*) of hot-pressed PPET-P increases significantly with the addition of the EMIM:TCM dopant. When the molar concentration of EMIM:TCM, *n* = 0 M, and the *σ* value of PPET_0_-P is 0.34 S/cm, which is 2.5 times less than that prepared by depositing the PEDOT:PSS onto glass substrate (PPET_0_-G), i.e., 0.85 S/cm. For *n* = 0.17 M, the *σ* of PPET-P shows a peak value of 62.57 S/cm, which is 184 times that of PPET_0_-P. A similar phenomenon was found in the PPET-G samples, as reported in our previous work [6]. This phenomenon could be rationalized with the fact that the EMIM:TCM doping has changed the quaternary structure of PEDOT:PSS [26], which leads to the formation of long-range ordered conductive paths [6,27].

Figure 2b shows that the *S* value of hot-pressed PPET-P decreases monotonically with the increase in *n*, i.e., *S* decreases from 42.2 to 27.2 μV K^−1^ when the *n* increases from 0 to 0.3 M. As a result, the PF value of hot-pressed PPET-P achieves a maximum value of 6.82 μW m^−1^ K^−2^ for *n* = 0.17 M (Figure 2c). By comparing with the literature reports, it is found that both the *S* and PF values of PPET_0.17_-P are higher than those reported in the previous work for paper-based TEGs, as shown in Figure 2d,e. And the detailed S, PF and conductivity values were given in the Table S1. It should be noted that the paper/CNT hybrids show higher *σ* values (Figure 2d) than PPET_0.17_-P due to the good conductivity of CNT. Moreover, for *n* = 0.17 M, the *σ* and PF values of PPET g (i.e., 1163 S/cm and 175 μW m^−1^ K^−2^, respectively) are much higher than those of PPET-P [27,28]. In the following section, the decent thermoelectric performance of hot-pressed PPET_0.17_-P will be discussed by combining with the structure information.

**Figure 3:**
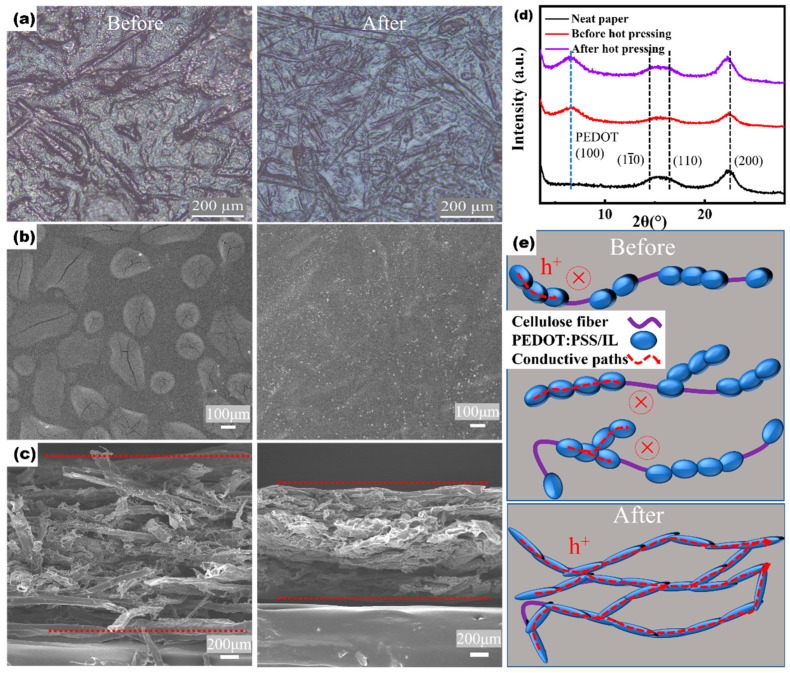
Structure and property correlation of PPET_0.17_-P. (**a**) Optical microscopy images of PPET-P before and after hot-pressing. (**b**) Plane view and (**c**) cross-sectional scanning electron microscopy data of PPET-P before and after hot-pressing. (**d**) GIXRD data. The (1ī0), (110), and (200) crystalline planes of the type I cellulose crystal and (100) crystalline plane of PEDOT crystal are indicated with dashed lines. (**e**) Proposed conductive paths (indicated with dashed-line arrows) in PPET-P before and after hot-processing. In the panel (**e**), the X mark circled around by dashed-line indicated the discontinuous conductive paths due to untouched PEDOT region.

To explore the decent thermoelectric performance of hot-pressed PPET_0.17_-P, the samples’ surface morphology and crystalline structure were characterized using optical microscopy (OM), scanning electron microscopy (SEM), and grazing-incidence X-ray diffraction (GIXRD), respectively. Based on these data, the structure–property correlation will be discussed. The OM data show that the samples’ surface are fully covered by PPET, indicated by the unique light blue feature of PEDOT:PSS (Figure 3a). Below the PPET, the rough features of cellulose fibers are observed, indicating the PPET attached well to the cellulose fibers. Compared to the PPET-G sample, the microstructural packing of PPET could be changed by the tomography of the paper substrate and their interactions with cellulose fibers. In addition, hot-pressing would improve the packing density of the cellulose fibers and also the attached PPET. Further surface structure information could be extracted from the SEM data. Figure 3b shows the plane-view SEM images of the PPET_0.17_-P before and after hot-pressing. Grain-like features are observed in the PPET_0.17_-P sample before hot-pressing, which could be attributed to the localized aggregation of PEDOT:PSS microdomains in the paper matrix. In contrast, a smooth surface structure is observed in the hot-pressed PPET_0.17_-P sample, which could be attributed to the improved packing density of cellulose fibers and associated PPET, i.e., the improved inter-grain connection of PEDOT. The improved inter-grain connection would enable the formation of continuous conductive paths [33]. This behavior could be further demonstrated by observing the cross-sections of the sample. Figure 3c shows the cross-sectional SEM images of PPET_0.17_-P before and after hot-pressing. The thickness of the PPET_0.17_-P film decreased from ~200 to ~120 μm after hot-pressing, which is consistent with the improved packing density in the film thickness direction. In addition, the porosity of the cellulose fibers decreases after hot-pressing. The improved packing density of fibers is expected to help the associated PEDOT:PSS to establish long-range ordered conductive paths [19,20]; thus, a higher *σ* value is achieved (Figure 2).

The crystalline structure of the PPET_0.17_-P samples were characterized with GIXRD. Figure 3d shows that the neat paper sample has three diffraction peaks with a diffraction angle 2*θ* at around 14.5°, 16.5°, and 22.5°, respectively. They are attributed to the (1ī0), (110), and (200) crystalline planes of the type I cellulose crystal. These crystalline features are attributed to the native cellulose [34]. Note that four polymorphs of the cellulose crystals have been reported, namely, cellulose I, cellulose II, cellulose III, and cellulose IV, and they have different unit cells [35]. The crystalline features of cellulose crystal are also found in the PPET_0.17_-P samples before and after hot-pressing, with no change of the peak position. This phenomenon could be rationalized with the fact that the mixing with PPET and hot-pressing did not change the crystalline structure of the cellulose. It is inferred that the interactions among PPET and cellulose fiber stay mainly in the surface layer of the cellulose fiber. These interactions are dominated by the formation of hydrogen bonding, which has been demonstrated by Deng et al. [21]. In addition, a new peak with 2*θ* = 6.6° is found in the PPET_0.17_-P sample, and hot-pressing does not change the peak position. This peak is attributed to the (100) crystalline plane of PEDOT crystals [36]. Thus, the hot-pressing does not change the crystalline structures of cellulose and PEDOT. It is supposed that the influence of paper substrate and hot-pressing on the thermoelectric performance of PPET mainly lies in the higher level, for example, long-range ordered conductive paths.

In this section, the structure–property correlation of the PPET-P sample is discussed by taking the substrate and hot-pressing effect into consideration. As demonstrated in the previous works, EMIM:TCM would interact strongly with the PEDOT:PSS via ion exchanges [37,38], which enables the structural rearrangement of PEDOT chains that form long-range ordered conductive paths when they are dried in glass or silicon substrates. The long-range ordered conductive paths of PPET-G have been revealed by the synchrotron radiation small-angle X-ray scattering and micro-Fourier transform infrared spectroscopy results, as demonstrated by Li et al. [4]. In contrast, PPET would penetrate into the pores of the paper substrate that results in the PEDOT chains and grains being scattered over in the thickness direction (Figure 3e). As a result, the PEDOT chains/grains could not pack densely with each other to form an efficient conductive path. Thus, the PPET-P samples have much lower *σ* and PF values than those of PPET-G. Moreover, this drawback could be alleviated by hot-pressing, which causes the dense packing of cellulose fibers and the associated PPET to smear off the grain boundaries in PPET-P (Figure 3b). A similar phenomenon has also been reported by Palaporn et al. [24]. As schematically illustrated in Figure 3e, the ionic conductive path is improved in the hot-pressed PPET-P sample.

**Figure 4:**
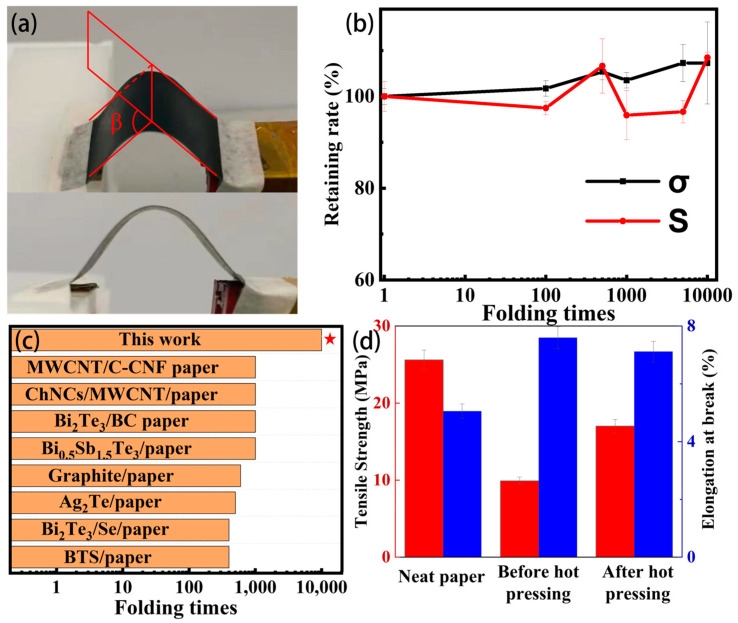
Mechanical behavior of hot-pressed PPET_0.17_-P. (**a**) Photo images of the paper sheet folded to an obtuse angle (*β* = 120°) by a home-made folding tester [39]. (**b**) Changes in *σ* and S of PPET_0.17_-P samples as a function of cyclic folding times. (**c**) Comparison with the literature reports about the maximum folding times of paper-based thermoelectric generators [25,27,29,31,40,41,42,43]. The value of this work was highlighted by a red star. (**d**) Tensile stress behavior of the neat paper substrate and PPET_0.17_-P sample before and after hot-pressing.

Mechanical properties including foldability and stretchability also play important roles in the paper-based thermoelectric and their applications in wearable electronics. This is because they determine the overall performance and stability of the flexible TEGs [5]. In this section, the mechanical properties of the hot-pressed PPET_0.17_-P are studied. The mechanical stability of the hot-pressed PPET_0.17_-P sample was evaluated using the cyclic folding test. Figure 4a shows the maximum folding angle (*β* = 120°) of the sample. For a typical folding cycle, the *β* value increases gradually from 0° to 120° and then returns to 0°, which is controlled by the slider of the tester. The *σ* and *S* values of the cyclically folded samples were collected, and the typical values are shown in Figure 4b. For clarity reason, the change in the *σ* and *S* values as a function of the cyclic folding times is indicated by the ratio of the cyclically folded value to the original one. Both *σ* and *S* values keep almost constant at 100%, indicating the superior folding stability of the hot-pressed PPET_0.17_-P samples. In addition, the maximum cycling times (10,000) used in this work is almost 10 times larger than that reported in the literature for paper-based thermoelectric generators (Figure 4c) while maintaining the integrity of the appearance (Figure S2), indicating unprecedent folding stability was achieved in the hot-pressed PPET_0.17_-P sample. Moreover, the stretchability of the hot-pressed PPET_0.17_-P sample was also measured. Figure 4d shows that the hot-pressed PPET_0.17_-P sample has tensile strength and elongation at break values of 17.5 MPa and 7.2%, respectively. These values are lying between those of the neat paper and the PPET_0.17_-P sample without pressing, indicating that a balanced stretching performance was achieved in the hot-pressed PPET_0.17_-P sample. These improved mechanical stabilities would be expected to endow the TEGs with better overall performance.

**Figure 5:**
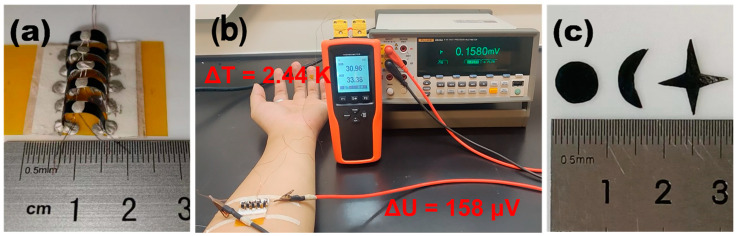
TEGs fabricated with PPET_0.17_-P. (**a**) Photon image of the PPET_0.17_-PTEG consisting of five pairs of legs made of PPET_0.17_-P. (**b**) Thermoelectric performance of the PPET_0.17_-PTEG. (**c**) PPET_0.17_-P cut into different size and shape.

To demonstrate the thermoelectric performance of the PPET-P in practical applications, the hot-pressed PPET_0.17_-P sheets were cut into rectangular shape and assembled into TEGs. Figure 5a shows the photon image of the PPET_0.17_-PTEG consisting of five couples of legs. The TEG device could be worn in the front arm (Figure 5b). The PPET_0.17_-PTEG generated an output voltage of 158 μV at a temperature difference of 2.44 K. Although this value is far below the minimum value required to power wearable electronics (~1 mV [44,45]), it could be increased by increasing the numbers of leg pairs and the temperature difference. However, these studies are out the scope of the present work. Meanwhile, the hot-pressed PPET_0.17_-P sheet also has the advantages of the paper substrate [13], which could be easily cut into different shapes (Figure 5c) such as sun, moon and star. This feature would enable the wide applications of PPET-P in the flexible electronics.

## 4. Conclusions

A paper-based thermoelectric material was successfully prepared by drop-casting combined with hot-pressing. The as-prepared PPET-P show decent thermoelectric performance, i.e., PF = 6.82 μW m^−1^ K^−2^ for PPET_0.17_-P, which is better than the reported performance for paper-based thermoelectric in the literature. This phenomenon was attributed to the synergistic effect of high-performance thermoelectric ink (PPET) and the post-treatment with hot-pressing. It has been demonstrated that the performance of PPET-P could be improved by hot-pressing via increasing the packing density of cellulose and associated PPET, which improved the inter-grain connection of PEDOT. Moreover, the PPET_0.17_-P sheet showed unprecedent folding stability with cyclic folding times as high as 10,000, and also good stretchability. Together with the good tailorability inherited from the paper substrate, the PPET_0.17_-P sheet can be fabricated into wearable TEGs, which are attractive for the potential applications in light-weight, environmentally friendly, and disposable wearable electronics.

## Data Availability

The data presented in this study are available on request from the corresponding author.

## Supplementary Materials

The following supporting information can be downloaded at: https://www.mdpi.com/article/10.3390/polym15214215/s1. Figure S1: The conversion mechanism of TEGs. Figure S2: Photograph of the sample before and after 10K cycles. Figure S3: Photograph of home-made bending performance testing device and the scheme of the home-made Seebeck coefficient measurement system. Table S1: Comparison with the literature reported *S*, PF and *σ* values of paper-based thermoelectric materials. Refs. [21,27,28,29,30,31,32] are cited in the supplementary materials.
