# Supplementary material for: Highly Flexible and Foldable Paper-Based Thermoelectric Generator Prepared with Post-Treatment-Free PEDOT:PSS Hybrid Ink
Source: Polymers (Basel). 2023 Oct 25;15(21):4215. doi: 10.3390/polym15214215 (PMC10647209; doi:10.3390/polym15214215)
Supplement: Supplementary file 1 [file polymers-15-04215-s001.zip › polymers-2641086-supplementary.pdf]

Supplementary Table S1. Comparison with the literature reported S, PF and σ values of paper-based thermoelectric materials

Authors	materials	S (μV/K)	PF ($\mu V{\cdot}m^{-1}\ {\cdot}K^{-2}$)	σ S/cm
Deng, L. et al.[1]	PEDOT:PSS/Xuan paper	20.5	0.97	22.13
Li, H. et al. [2]	MWCNT/C-CNF paper	12.7	2.06	128
Miyama, A. et al. [3]	CNT/paper	22.6	5.1	99.9
He, Y. et al. [4]	ChNCs/MWCNT/paper	20	0.46	11.5
Brus, V. V. et al. [5]	PEDOT:PSS/paper	24.8	0.0726	1.18
Mulla, R. et al. [6]	PEI/graphite/paper	-21.5	0.1	2.24
Li, J. et al. [7]	PEDOT:PSS/PPy/paper	16	0.00934	0.365
This work	PEDOT:PSS/EMIM:TCM/paper	33	6.82	62.57

Supplementary Text

Supplementary figure S1 shows the conversion mechanism from thermal energy to electrical energy. It's a conventional π-shape thermoelectric (TE) model consist of two TE legs made by p-type and n-type materials. The charge carriers (hole or electron) will undergo directional movement (diffusion from the hot end to the cold end) at a temperature gradient, resulting in a potential difference and achieving TE power generation.

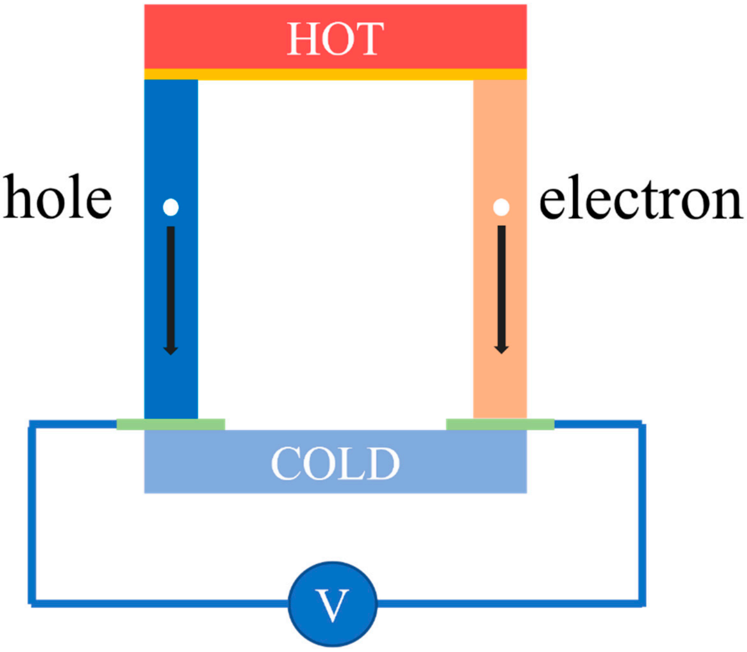

Supplementary figure S1. The mechanism of thermoelectric power generation

Supplementary figure S2 shows the photographs of the $PPET_{0.17}$-P sample before and after 10000 cycles. After external pressure stimuli, the sample only slightly wrinkles, indicating high mechanical properties of paper-based flexible TEGs.

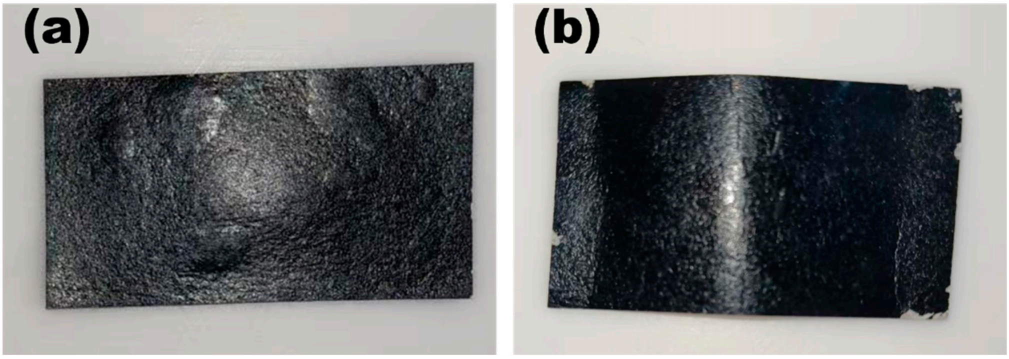

Supplementary figure S2. The photographs of $PPET_{0.17}$-P before (a) and after (b) 10000 bending cycles

Supplementary Methods

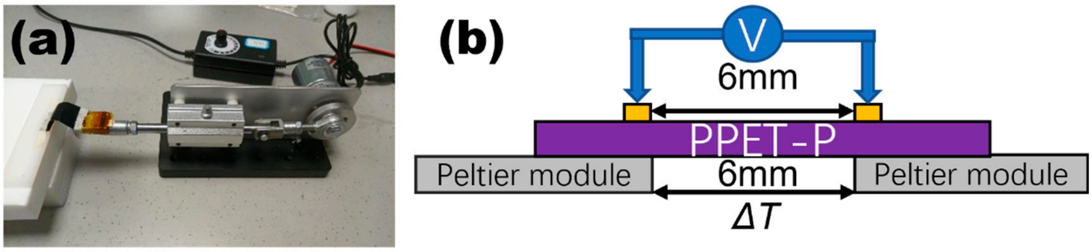

Supplementary figure S3. (a) the photograph of home-made bending performance testing device. (b) Scheme of the home-made Seebeck coefficient measurement system.

Supplementary Fig. S3(a) shows the testing scenario for the bending performance of the sample, with the motor rotating and driving the rod to move, causing the sample of PPET-P to bend. As shown in Supplementary Fig. S3(b), the thermocouples were placed on top of the PPET-P with a gap of 6 mm and used to collect both T and V data. The temperature differences along the line of the two probes were controlled by the Peltier temperature modules.

Reference

1 Deng, L.; Zhang, Y.; Wei, S.; Lv, H.; Chen, G. Highly foldable and flexible films of PEDOT:PSS/Xuan paper composites for thermoelectric applications. *Journal of Materials Chemistry A* **2021**, 9, 8317-8324.

2 Li, H.; Zong, Y.; Ding, Q.; Han, W.; Li, X. Paper-based thermoelectric generator based on multi-walled carbon nanotube/carboxylated nanocellulose. *Journal of Power Sources* **2021,** *500*, 229992.

3 Miyama, A.; Oya, T. Improved performance of thermoelectric power generating paper based on carbon nanotube composite papers. *Carbon Trends* **2022,** *7*, 100149.

4 He, Y.; Lin, X.; Feng, Y.; Luo, B.; Liu, M. Carbon Nanotube Ink Dispersed by Chitin Nanocrystals for Thermoelectric Converter for Self-Powering Multifunctional Wearable Electronics. *Advanced Science* **2022,** *9*, 2204675.

5 Brus, V. V.; Gluba, M.; Rappich, J.; Lang, F.; Maryanchuk, P. D.; Nickel, N. H. Fine Art of Thermoelectricity. *ACS Applied Materials & Interfaces* **2018,** *10*, 4737-4742.

6 Mulla, R.; Jones, D. R.; Dunnill, C. W. Thermoelectric Paper: Graphite Pencil Traces on Paper to Fabricate a Thermoelectric Generator. *Advanced Materials Technologies* **2020,** *5*, 2000227.

7 Li, J.; Du, Y.; Jia, R.; Xu, J.; Shen, S. Z. Thermoelectric Properties of Flexible PEDOT:PSS/Polypyrrole/Paper Nanocomposite Films *Materials* [Online], 2017, p. 780.
